# Impact of Smoking on Overall and Cancer-Specific Mortality in Prostate Cancer: Elevated Risks in Older and Early-Stage Patients—A Population-Based Study

**DOI:** 10.3390/life14101281

**Published:** 2024-10-09

**Authors:** Ren-Jie Lin, Chien-Liang Liu, Steven K. Huang, Allen W. Chiu, Yu-Cih Wu, Wen-Hsin Tseng, Chung-Han Ho

**Affiliations:** 1 Department of General Medicine, Chi Mei Medical Center, Tainan 710402, Taiwan; 0440lsda@gmail.com; 2 Division of Urology, Department of Surgery, Chi Mei Medical Center, Tainan 710402, Taiwan; bearlau.tw@gmail.com (C.-L.L.); skhsteven@yahoo.com.tw (S.K.H.); 3 Department of Urology, Shin Kong WHS Memorial Hospital, Taipei 111045, Taiwan; whchiu1216@gmail.com; 4 Department of Medical Research, Chi Mei Medical Center, Tainan 710402, Taiwan; 5 Institute of Biomedical Science, National Sun Yat-sen University, Kaohsiung 804201, Taiwan; 6 Department of Information Management, Southern Taiwan University of Science and Technology, Tainan 710301, Taiwan; 7 Cancer Center, Taipei Municipal Wanfang Hospital, Taipei Medical University, Taipei 116079, Taiwan

**Keywords:** prostate cancer, smoking, Taiwan Cancer Registry, cancer stages

## Abstract

Prostate cancer (PCa) ranks sixth in cancer mortality among Taiwanese men, with smoking rates remaining high despite the 2009 Tobacco Hazards Prevention Act. This study used the Taiwan Cancer Registry to evaluate smoking’s impact on PCa mortality, providing important information for healthcare strategies and patient management. From 2011 to 2017, 23,107 PCa patients were analyzed, with 7164 smokers and 15,943 non-smokers. The baseline characteristics, clinical stages, comorbidities, and treatment modalities were all included to estimate overall and cancer-specific mortality using the Cox regression model and Kaplan–Meier analysis. The stratified analysis of clinical stage and age group was also estimated. Our study found an association between smoking and increased overall and cancer-specific mortality in PCa patients. Although smokers over 60 had higher risks of overall mortality than non-smokers, cancer-specific mortality did not show significant differences in any age group. Smokers had higher overall mortality than non-smokers across all clinical stages, but cancer-specific mortality was significantly raised only in early-stage cases. In conclusion, smoking is associated with higher overall mortality in PCa patients, with a significant increase in cancer-specific mortality in early-stage cases. Therefore, active smoking management is critical for clinical urologists, particularly in the treatment of early-stage patients.

## 1. Introduction

Prostate cancer (PCa) is the second most common cancer worldwide and the fifth leading cause of cancer mortality among men, accounting for 7.3% of new cases and 3.8% of deaths from all cancers in 2020 [1]. In Taiwan, according to cancer registration statistics for 2021, PCa ranked fifth in incidence among the top ten cancers, with 7500 new cases reported. Additionally, it was the sixth leading cause of cancer deaths, with a crude mortality rate of 14.59 per 100,000 population [2]. Compared to 2012, both the mortality rate and incidence of prostate cancer have increased, with mortality rising from 10.17 to 14.59 per 100,000 population and incidence from 40.61 to 64.77 per 100,000 population [2,3]. A recent study found that the increasing use of prostate-specific antigen (PSA) tests and metabolic syndrome-related diseases might be associated with the increasing incidence of PCa [4].

For most patients, definitive treatments for localized PCa are mainly radiotherapy or radical prostatectomy, often followed by androgen deprivation therapy (ADT) or active surveillance. The treatment recommendations are mostly based on the risk group stratified by Gleason score, PSA, and clinical T stage [5]. Various risk factors have been confirmed to be associated with the mortality of prostate cancer. These include non-modifiable factors such as race, family history, and germline mutations (BRCA2, BRCA1, CHEK2, MSH2, MSH6, EZH2, LSD1…) [6,7], and modifiable factors such as meat consumption and trans fat acid intake [8]. However, for several other modifiable risk factors, their association with prostate cancer remains inconclusive. Among these, smoking is an important and common risk for its association with the mortality of several genitourinary malignancies, such as bladder cancer (BC) [9,10], upper tract urothelial carcinoma (UTUC) [11], and renal cell carcinoma (RCC) [12]. However, research results regarding its relationship with PCa mortality remain inconsistent. Although a few studies have suggested that smoking may be associated with lethal PCa, most studies revealed no significant association [13,14,15]. In another meta-analysis, smokers were even found to have a significantly lower risk of PCa [16]. Although this result was hypothesized to be related to poor compliance, further research is needed to confirm the association between smoking and PCa [16,17]. Furthermore, research on this association in Asian populations is still lacking. In Taiwan, despite the enactment of the Tobacco Hazards Prevention Act in 2009, the prevalence of smoking among adult males still reached 23.1% in 2021 [18]. Therefore, confirming the association between smoking and PCa mortality would aid in treatment and lifestyle recommendations, as well as clinical risk assessments.

Taiwan’s National Health Insurance Research Database (NHIRD) was used to investigate the relationship between smoking and both overall mortality and cancer-specific mortality in PCa patients within the Taiwanese population. Additionally, we conducted further stratified analyses on related risk factors.

## 2. Materials and Methods

### 2.1. Data Source

This study utilized data from the Taiwan Cancer Registry (TCR), the National Health Insurance Research Database (NHIRD), and the Cause-of-Death Database, all of which were sourced from the Health and Welfare Data Science Center (HWDC), an integrated hub for health-related datasets. The TCR was established in 1979 to monitor Taiwan’s cancer incidence and mortality rates. It includes information on individual demographics, cancer stages, primary cancer sites, tumor histology, and treatment types. The NHIRD is based on Taiwan’s national health insurance program, which includes detailed healthcare information about more than 99% of Taiwan’s population from 1996 to 2018. For research purposes, the HWDC released the de-identified claims to the public in an anonymous format. This study was conducted in compliance with the Declaration of Helsinki and has been approved by the Research Ethics Committee of Chi Mei Hospital (IRB no. 11309-002). In addition, patient informed consent was waived by the Research Ethics Committee of Chi Mei Hospital.

### 2.2. Definitions of Study Subjects

**Figure 1:**
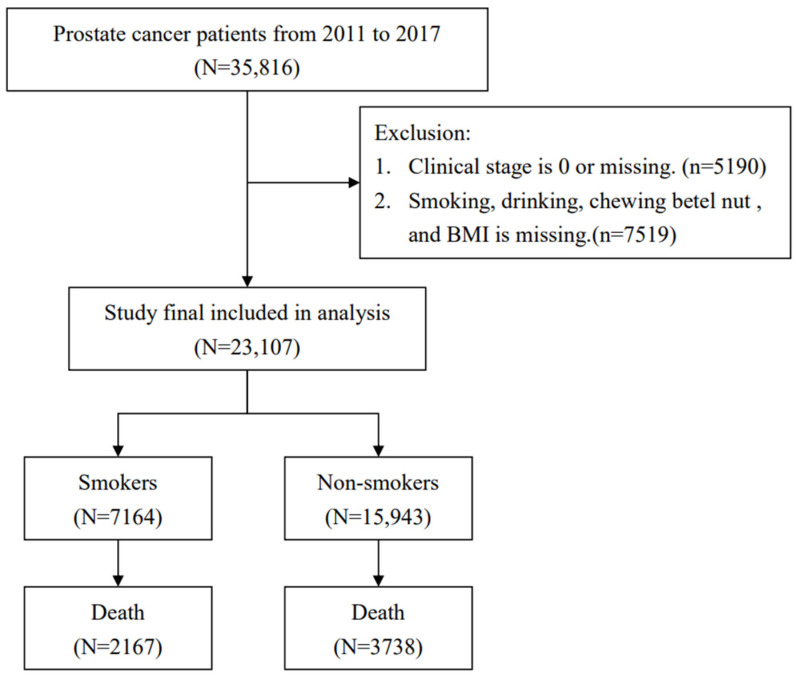
Flowchart of study subjects’ selection.

Patients with prostate cancer between 2011 and 2017 were selected from the TCR using the International Classification of Diseases for Oncology, third edition (ICD-O-3): C61. The patients with incomplete information in the TCR were excluded from this study. A flowchart summarizing the selection of study subjects is presented in Figure 1.

### 2.3. Measurement

The primary outcome of this study was mortality. Mortality was determined from the Cause-of-Death Dataset. The mortality risk was estimated using two endpoints: overall and cancer-specific mortalities. The time for the overall mortality was set from the date of the prostate cancer diagnosis to the date of death, regardless of the cause. Cancer-specific mortality was defined as the cause of death due to prostate cancer. For estimating the association between mortality and underlying diseases, all enrolled study subjects were linked with NHIRD to collect the diagnoses records.

The Charlson’s Comorbidity Index (CCI) scores were calculated as the disease severity levels, which were divided into three disease severity groups: 0, 1–2, and ≥3. Diabetes mellitus, hypertension, and hyperlipidemia were also considered in this study. Patients with comorbidities were defined as patients with the diseases included below at least one year before the diagnosis of prostate cancer. The definition of the above comorbidities was based on the International Classification of Diseases, Ninth Revision, Clinical Modification (ICD-9-CM) codes from 2011 to 2014 or the International Classification of Diseases, Tenth Revision, Clinical Modification diagnostic codes after 2015, given in Supplementary Materials.

### 2.4. Statistical Analysis

The baseline information of categorical variables is presented as frequencies with percentages. The distribution difference between smokers and non-smokers was compared using Pearson’s chi-square test. Additionally, the trend of mortality risk was plotted using the Kaplan–Meier approach with the log-rank test to compare the trend difference. The Cox proportional regression model was used to estimate the hazard ratios (HRs) of the overall and cancer-specific mortalities. Adjusted HRs were calculated from the multivariable Cox regression model, with adjustment for age at diagnosis, sex, body mass index (BMI) score, Charlson Comorbidity Index (CCI) score, drinking, whether the patient chews betel nuts, clinical stage, treatment type, and comorbidity. The stratified analysis was also presented to estimate the risk ratios of the overall and cancer-specific mortalities between smokers and non-smokers with prostate cancer in different clinical stages and age groups. To avoid violating the proportional hazards assumption, the estimated HRs were checked using the Schoenfeld residuals test. The SAS 9.4 (SAS Institute, Inc., Cary, NC, USA) was used to perform all statistical analyses. Kaplan–Meier curves were plotted using Stata version 12 (Stata Corp., College Station, TX, USA). A *p*-value < 0.05 was set for statistical significance.

## 3. Results

A total of 23,107 patients were included in this study, with 15,943 non-smokers and 7164 smokers. Table 1 presents the characteristics of non-smokers and smokers with prostate cancer. In the age distribution, smokers were generally younger, with a higher proportion under 60 and a lower proportion ≥80 compared to non-smokers (9.56% vs. 6.65% and 21.83% vs. 25.13%, respectively) (*p* < 0.0001). More smokers were diagnosed at stages 3 or 4 (14.24% vs. 13.76% and 35.71% vs. 30.60%) and fewer at stages 1 or 2 (10.12% vs. 12.12% and 39.94% vs. 43.53%) compared to non-smokers (*p* < 0.0001). Non-smokers had a higher proportionality in terms of CCI score, with one of 0 (27.65% vs. 24.26%), whereas smokers had greater levels, with CCI scores of ≥3 (38.37% vs. 33.71%) (*p* < 0.0001). Diabetes (DM), hyperlipidemia, and hypertension (HTN) showed similar distributions between groups. Smokers were more likely to drink alcohol (46.30% vs. 9.71%, *p* < 0.0001) and chew betel nuts (15.83% vs. 0.78%, *p* < 0.0001). A lower proportion of non-smokers had a BMI < 18.5 (3.79% vs. 5.58%), but BMI ≥ 25 had similar proportionality (41.54% vs. 42.02%). A higher proportion of non-smokers underwent surgery (55.95% vs. 52.58%, *p* < 0.0001), while more smokers received radiotherapy and hormone therapy (28.96% vs. 27.30%, *p* = 0.0092 and 66.00% vs. 60.63%, *p* < 0.0001). Overall mortality and prostate cancer-specific mortality were higher in smokers (30.25% vs. 23.45%, *p* < 0.0001 and 14.94% vs. 12.26%, *p* < 0.0001).

**Figure 2:**
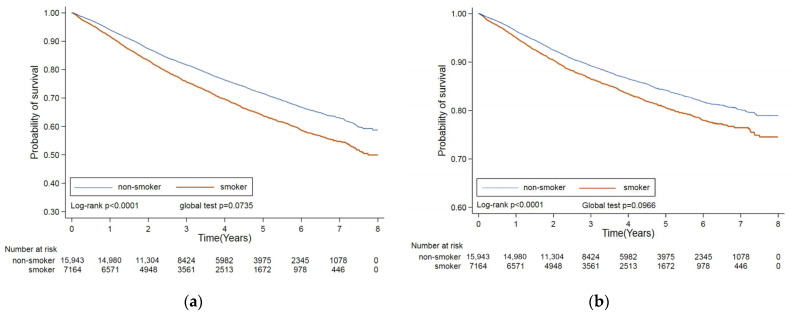
Association of cigarette smoking with mortality risk of prostate cancer: (**a**) overall mortality and (**b**) cancer-specific mortality.

The Kaplan–Meier curves for overall mortality (a) and cancer-specific mortality (b) presented the trends between the non-smokers and smokers and were illustrated in Figure 2. Smokers were associated with poor overall survival (*p* < 0.0001) as well as cancer-specific mortality (*p* < 0.0001) compared with non-smokers during the study periods. Additionally, the estimated overall and cancer-specific mortality risks between smokers and non-smokers among prostate cancer patients are presented in Table 2. Compared to non-smokers, smokers had 1.27 folds of overall (95% CI: 1.19–1.35; *p* < 0.0001) and 1.12 folds of cancer-specific mortality (95% CI: 1.03–1.22; *p* = 0.0067) after adjustment for selected confounding factors.

Table 3 shows stratified analysis by age groups and clinical stages. The effect of smoking on mortality was not prominent in those who were under 60 in age (*p* = 0.6101). Although smokers had higher overall and cancer-specific mortality risks than non-smokers, the cancer-specific mortality association diminished in the 60–70, 70–80, and ≥80 age groups after stratification (*p* = 0.5371, 0.0629, and 0.0913). Across all clinical stages, smokers had higher overall mortality risks. The association with cancer-specific mortality was only significantly higher in stage II after stratification (*p* = 0.0250). This result was still significant in the combined effect of stage I and stage II (early-stage group, *p* = 0.0139).

## 4. Discussion

As a modifiable risk factor for PCa, the impact of smoking on PCa may vary depending on the patient’s race [19]. Standardized by age, Northern Europe had the highest incidence of PCa, while the Caribbean had the highest mortality rate. In contrast, populations in South–Central Asia had the lowest incidence and mortality rate of PCa [19]. Some studies have further explored the association between smoking and the aggressiveness of PCa across different populations. A cross-sectional study among African Americans and European Americans revealed that current smokers had 1.99 times the odds of developing highly aggressive PCa compared to people who had never smoked. After stratifying by race, higher odds of highly aggressive PCa were still observed in African Americans, but not in European Americans [20]. In another cross-sectional study in Italy, smoking status also failed to demonstrate an association with both diagnosis of PCa or high-grade PCa [21]. Similarly, in a retrospective study in South Nigeria, although the results were not statistically significant, smokers were more commonly diagnosed with Gleason score ≥ 8 PCa [22]. Except for the oncological outcomes, smokers were also associated with poorer functional outcomes in patients with localized PCa that underwent radical prostatectomies. Postoperatively, they were associated with poorer erectile function that might improve after 18 months of smoking cessation [23].

In the present study, we analyzed the association between smoking status and both overall mortality and cancer-specific mortality in patients with prostate cancer. The result showed that, compared to non-smokers, smokers had 27% and 12% increases in risks of overall and prostate cancer-specific mortality (PCSM), respectively. Furthermore, the age-stratified analysis revealed that the impact of smoking on mortality was not significant in patients under 60 years old (*p* = 0.68 and 0.61 for overall and cancer-specific mortality, respectively). For patients aged 60–70, 70–80, and over 80, smoking increased the adjusted HRs for overall mortality by approximately 1.23–1.29, but there was no significant effect on cancer-specific mortality. These results suggest that smoking might more significantly affect the general physical health of individuals, leading to all-cause mortality, rather than directly contributing to the progression of prostate cancer itself across different age groups. Therefore, the impact of smoking on overall mortality was not significant in the age < 60 group, which had a relatively better physical health status, but showed a significant effect in older age groups. In our review, older patients tended to experience increased oncological risk, and similar findings have been observed in bladder cancer [24].

In clinical stage-stratified analysis, smokers had higher risks of overall mortality across all stages. However, only smokers in early stages were associated with a higher risk of PCSM (adjusted HR = 1.35, *p* = 0.0139). In the previous study, smokers were associated with poorer compliance, which might explain our results. Even if palpable nodules in direct rectal examination (DRE), elevated PSAs, or a Gleason score of 7 or 8 in biopsies were noted, delayed diagnoses and poor treatment compliance could result in higher PCSM in patients with early stage PCa [25,26].

For PCa patients treated with RP, Moreira et al. reported that smokers were associated with higher risks of metastasis and overall mortality, but no association with PCSM was noted [27]. In another cohort treated with RT, a similar association between smoking and distant metastasis, but not with prostate cancer-specific death, was reported [28]. These findings were incorporated into a meta-analysis by Foerster et al., where the pooled results revealed that smokers had higher risks of biochemical recurrence (BCR), metastasis, and cancer-specific mortality after RT or RP [14]. However, many of these studies were conducted in Western cohorts. In the Asian experience, Oh et al. found that smokers were not associated with worse BCR or pathological outcomes after RP [29]. These findings indicate that cultural differences and lifestyles between the Eastern and Western worlds might also influence treatment outcomes in PCa patients.

Our results also found that a CCI ≥ 3 was associated significantly with increased risks of overall mortality and PCSM, 87% and 47% respectively. In another Taiwan population-based study, Shih et al. reported that a CCI > 3 was associated with overall mortality and PCSM, particularly in patients within low or intermediate risk groups [30]. For patients with high, very high, and metastatic risks of PCa, the association between a CCI > 3 and PCSM was not observed in their analyses.

Furthermore, a BMI ≥ 25 and hyperlipidemia appeared to be protective factors in our analyses. As shown in Table 2, patients with BMI ≥ 25 were associated with lower risks of OS and PCSM compared to those with BMIs between 18 and 25. Currently, the “obesity paradox” supports the phenomenon of the survival benefits associated with the risks of obesity. In an analysis of 1577 men with metastatic castration-resistant prostate cancer (mCRPC), Martini et al. found a protective effect of obesity (BMI > 30) on overall mortality and PCSM. This association was not influenced by a dose of docetaxel, and the underlying mechanism remained unclear. Similar observations have been also made in other genitourinary cancers, such as RCC and BC [31,32]. Further research is warranted to elucidate this paradox.

Regarding hyperlipidemia, 17% and 14% reductions in the risk of overall mortality and PCSM were noted in our analysis, as shown in Table 2. Due to the retrospective nature from Taiwan’s National Health Insurance Research Database, the information we obtained was merely the diagnosis of hyperlipidemia, instead of the associated information such as, lipid profile, medication used, and dynamic change in lipid profile during the treatment duration. However, a study on statin utilization in Taiwan by Hsieh et al. reported that approximately 77% of statin users were diagnosed with dyslipidemia in 2011, reflecting a high utilization rate of statins to treat hyperlipidemia in the Taiwanese population [33]. Therefore, the relationship between hyperlipidemia and prostate cancer survival benefits might result from the high utilization rate of statins, suggesting that hyperlipidemia acts as a confounding factor. Corresponding to this hypothesis, a prospective study by Geybels et al. reported the association between statin use and the reduced PCSM [34]. Additionally, Van Rompay et al. further demonstrated this protective effect with non-statin lipid-lowering agents [35].

There are some limitations to our study. First, all of our data were retrospectively retrieved from a nationwide database, which limited our ability to obtain detailed smoking information, such as smoking duration, pack-years, time of cessation, and changes in smoking habits after the diagnosis and treatment of prostate cancer. Additionally, detailed cancer information, including histological type, PSA, Gleason score, magnetic resonance image (MRI) description, and tumor recurrence, could not be fully obtained. In the future research, we believe that the Gleason score in combination with multiparametric MRIs and the Prostate health index can be helpful in evaluating the risk of PCa and enabling early diagnosis and treatment for patients [36]. Second, our analysis was restricted to the Taiwanese population, reflecting a relatively regional result within Asia. This may limit the generalizability of our findings to other populations. Lastly, due to the retrospective nature of our study, the potential selection and information biases may affect the accuracy of our results; however, the large sample size could help reduce these biases.

## 5. Conclusions

In conclusion, our study demonstrated an association between smoking and increased overall mortality in PCa patients older than 60 across all clinical stages. Furthermore, smoking was associated with increased PCSM in early-stage prostate cancer. Other identified risk factors included a CCI > 3. Additionally, a diagnosis of hyperlipidemia and a BMI > 25 seemed to be protective factors in this study. The active management of smoking, comorbidities, and obesity can be helpful for urologists in treating prostate cancer, particularly in early-stage patients.

## Figures and Tables

**Table 1:** The characteristics of smokers and non-smokers among patients with prostate cancer.

Characteristic	Overall (N = 23,107)	Non-Smokers (N = 15,943)	Smokers (N = 7164)	*p*-Value
Age group				
<60	1745 (7.55)	1060 (6.65)	685 (9.56)	<0.0001
60–70	6923 (29.96)	4736 (29.71)	2187 (30.53)	
70–80	8868 (38.38)	6140 (38.51)	2728 (38.08)	
≥80	5571 (24.11)	4007 (25.13)	1564 (21.83)	
Clinical stage				
1	2657 (11.5)	1932 (12.12)	725 (10.12)	<0.0001
2	9801 (42.42)	6940 (43.53)	2861 (39.94)	
3	3213 (13.9)	2193 (13.76)	1020 (14.24)	
4	7436 (32.18)	4878 (30.60)	2558 (35.71)	
CCI				
0	6147 (26.6)	4409 (27.65)	1738 (24.26)	<0.0001
1–2	8837 (38.24)	6160 (38.64)	2677 (37.37)	
≥3	8123 (35.15)	5374 (33.71)	2749 (38.37)	
Drinking	4865 (21.05)	1548 (9.71)	3317 (46.30)	<0.0001
Chewing	1258 (5.44)	124 (0.78)	1134 (15.83)	<0.0001
Treatment				
Operation	12,687 (54.91)	8920 (55.95)	3767 (52.58)	<0.0001
Radiotherapy	6428 (27.82)	4353 (27.30)	2075 (28.96)	0.0092
Hormone	14,394 (62.29)	9666 (60.63)	4728 (66.00)	<0.0001
Comorbidity				
DM	5529 (23.93)	3758 (23.57)	1771 (24.72)	0.0582
Hyperlipidemia	5687 (24.61)	3886 (24.37)	1801 (25.14)	0.2117
HTN	12,829 (55.52)	8918 (55.94)	3911 (54.59)	0.0572
BMI				
<18.5	1005 (4.35)	605 (3.79)	400 (5.58)	<0.0001
18.5–25	12,469 (53.96)	8715 (54.66)	3754 (52.40)	
≥25	9633 (41.69)	6623 (41.54)	3010 (42.02)	
Death	5905 (25.56)	3738 (23.45)	2167 (30.25)	<0.0001
Death in PC	3024 (13.09)	1954 (12.26)	1070 (14.94)	<0.0001

**Table 2:** The risk of overall and cancer-specific mortality among prostate cancer patients.

		Overall Mortality					Cancer-Specific Mortality				
	Total	Death, N (%)	Crude HR (95%CI)	*p*-Value	Adjusted HR (95%CI)	*p*-Value	Death in PC, N(%)	Crude HR (95%CI)	*p*-Value	Adjusted HR (95%CI)	*p*-Value
Smoking											
Non-smokers	15,943	3738 (23.45)	Ref.		Ref.		1954 (12.26)	Ref.		Ref.	
Smokers	7164	2167 (30.25)	1.34 (1.27–1.42)	<0.0001	1.27 (1.19–1.35)	<0.0001	1070 (14.94)	1.26 (1.17–1.36)	<0.0001	1.12 (1.03–1.22)	0.0067
Age group											
<60	1745	213 (12.21)	Ref.		Ref.		164 (9.4)	Ref.		Ref.	
60–70	6923	922 (13.32)	1.17 (1.01–1.35)	0.0423	1.11 (0.96–1.29)	0.1581	558 (8.06)	0.91 (0.76–1.08)	0.2803	0.89 (0.75–1.06)	0.1926
70–80	8868	2145 (24.19)	2.18 (1.89–2.51)	<0.0001	1.72 (1.49–1.99)	<0.0001	1033 (11.65)	1.35 (1.14–1.59)	0.0004	1.06 (0.90–1.25)	0.4943
≥80	5571	2625 (47.12)	5.20 (4.52–5.98)	<0.0001	2.97 (2.57–3.43)	<0.0001	1269 (22.78)	3.17 (2.69–3.73)	<0.0001	1.66 (1.40–1.96)	<0.0001
Clinical stage											
1	2657	296 (11.14)	Ref.		Ref.		38 (1.43)	Ref.		Ref.	
2	9801	1497 (15.27)	1.42 (1.25–1.61)	<0.0001	1.24 (1.09–1.40)	0.0013	334 (3.41)	2.46 (1.76–3.43)	<0.0001	2.19 (1.56–3.07)	<0.0001
3	3213	527 (16.4)	1.51 (1.31–1.75)	<0.0001	1.26 (1.08–1.47)	0.0027	152 (4.73)	3.39 (2.38–4.84)	<0.0001	2.85 (1.97–4.11)	<0.0001
4	7436	3585 (48.21)	6.22 (5.52–7.01)	<0.0001	3.76 (3.29–4.30)	<0.0001	2500 (33.62)	32.96 (23.92–45.41)	<0.0001	20.71 (14.76–29.07)	<0.0001
CCI											
0	6147	1074 (17.47)	Ref.		Ref.		682 (11.09)	Ref.		Ref.	
1–2	8837	1892 (21.41)	1.28 (1.18–1.38)	<0.0001	1.20 (1.11–1.29)	<0.0001	956 (10.82)	1.01 (0.91–1.11)	0.8613	1.03 (0.93–1.14)	0.5558
≥3	8123	2939 (36.18)	2.49 (2.32–2.67)	<0.0001	1.87 (1.74–2.02)	<0.0001	1386 (17.06)	1.82 (1.66–1.99)	<0.0001	1.47 (1.33–1.63)	<0.0001
Comorbidity											
DM	5529	1609 (29.1)	1.27 (1.20–1.35)	<0.0001	1.11 (1.04–1.18)	0.0013	756 (13.67)	1.12 (1.03–1.22)	0.0060	1.08 (0.99–1.18)	0.1032
Hyperlipidemia	5687	1183 (20.8)	0.77 (0.72–0.82)	<0.0001	0.83 (0.77–0.88)	<0.0001	580 (10.2)	0.73 (0.66–0.80)	<0.0001	0.86 (0.78–0.94)	0.0016
HTN	12,829	3525 (27.48)	1.23 (1.17–1.30)	<0.0001	1.08 (1.02–1.15)	0.0052	1699 (13.24)	1.06 (0.99–1.14)	0.0978	1.07 (0.99–1.15)	0.1139
Drinking	4865	1291 (26.54)	1.02 (0.96–1.09)	0.4457	1.01 (0.94–1.08)	0.8623	659(13.55)	1.03 (0.94–1.12)	0.5691	1.01 (0.92–1.11)	0.8507
Chewing	1258	340 (27.03)	1.11 (0.99–1.24)	0.0666	1.04 (0.93–1.18)	0.8623	173(13.75)	1.10 (0.94–1.28)	0.2406	0.97 (0.82–1.14)	0.7147
Treatment											
Operation	12,687	2167 (17.08)	0.43 (0.41–0.45)	<0.0001	0.76 (0.72–0.81)	<0.0001	916 (7.22)	0.32 (0.30–0.35)	<0.0001	0.75 (0.69–0.81)	<0.0001
Radiotherapy	6428	1439 (22.39)	0.78 (0.74–0.83)	<0.0001	0.80 (0.75–0.85)	<0.0001	691 (10.75)	0.73 (0.67–0.79)	<0.0001	0.81 (0.74–0.88)	<0.0001
Hormone	14,394	4931 (34.26)	3.41 (3.19–3.66)	<0.0001	1.32 (1.21–1.44)	<0.0001	2768 (19.23)	7.24 (6.37–8.23)	<0.0001	1.33 (1.14–1.55)	0.0003
BMI											
<18.5	1005	579 (57.61)	2.94 (2.69–3.21)	<0.0001	2.02 (1.85–2.21)	<0.0001	338 (33.63)	3.22 (2.86–3.61)	<0.0001	2.23 (1.98–2.52)	<0.0001
18.5–25	12,469	3497 (28.05)	Ref.		Ref.		1814 (14.55)	Ref.		Ref.	
≥25	9633	1829 (18.99)	0.64 (0.60–0.68)	<0.0001	0.76 (0.72–0.80)	<0.0001	872 (9.05)	0.59 (0.54–0.64)	<0.0001	0.72 (0.67–0.78)	<0.0001

**Table 3:** The risk of overall and cancer-specific mortality among prostate cancer patients stratified by age groups and clinical stage.

		Overall Mortality					Cancer-Specific Mortality				
	Total	Death, N(%)	Crude HR (95%CI)	*p*-Value	Adjusted HR (95%CI)	*p*-Value	Death in PC, N(%)	Crude HR (95%CI)	*p*-Value	Adjusted HR (95%CI)	*p*-Value
Overall, smokers vs. non-smokers											
Smokers	7164	2167 (30.25)	1.34 (1.27–1.42)	<0.0001	1.27 (1.19–1.35)	<0.0001	1070 (14.94)	1.26 (1.17–1.36)	<0.0001	1.12 (1.03–1.22)	0.0067
Stratified											
Age group, smokers vs. non-smokers											
<60	685	112 (16.35)	1.74 (1.33–2.27)	<0.0001	1.07 (0.79–1.44)	0.6796	88 (12.85)	1.81 (1.33–2.47)	0.0001	1.09 (0.78–1.54)	0.6101
60–70	2187	394 (18.02)	1.66 (1.46–1.89)	<0.0001	1.23 (1.06–1.43)	0.0072	219 (10.01)	1.44 (1.21–1.70)	<0.0001	1.06 (0.88–1.29)	0.5371
70–80	2728	823 (30.17)	1.48 (1.36–1.62)	<0.0001	1.29 (1.17–1.43)	<0.0001	383 (14.04)	1.40 (1.23–1.59)	<0.0001	1.15 (0.99–1.32)	0.0629
≥80	1564	838 (53.58)	1.32 (1.22–1.43)	<0.0001	1.25 (1.14–1.37)	<0.0001	380 (24.30)	1.19 (1.05–1.34)	0.0051	1.12 (0.98–1.28)	0.0913
Clinical stage, smokers vs. non-smokers											
Stage I	725	103 (14.21)	1.46 (1.15–1.85)	0.0020	1.62 (1.24–2.12)	0.0004	13 (1.79)	1.43 (0.73–2.79)	0.2993	1.79 (0.85–3.75)	0.1236
Stage II	2861	548 (19.15)	1.45 (1.31–1.61)	<0.0001	1.38 (1.23–1.56)	<0.0001	116 (4.05)	1.34 (1.07–1.68)	0.0111	1.34 (1.04–1.72)	0.0250
Stage III	1020	212 (20.78)	1.42 (1.20–1.70)	<0.0001	1.57 (1.29–1.91)	<0.0001	51 (5.00)	1.07 (0.76–1.50)	0.6981	1.29 (0.89–1.86)	0.1802
Stage IV	2558	1304 (50.98)	1.12 (1.05–1.20)	0.0008	1.15 (1.06–1.24)	0.0006	890 (34.79)	1.09 (1.01–1.18)	0.0499	1.08 (0.99–1.19)	0.0871
Early stage	3586	651 (18.15)	1.46 (1.33–1.61)	<0.0001	1.42 (1.27–1.58)	<0.0001	129 (3.60)	1.36 (1.10–1.68)	0.0048	1.35 (1.06–1.71)	0.0139
Last stage	3578	1516 (42.37)	1.18 (1.11–1.26)	<0.0001	1.20 (1.11–1.28)	<0.0001	941 (26.30)	1.11 (1.03–1.20)	0.0092	1.09 (1.00–1.20)	0.0527

## Data Availability

The data sources are the Taiwan Nation Health Insurance Database and Taiwan Cancer Registry. The data are available with permission from the Taiwan Health and Welfare Data Science Center (https://dep.mohw.gov.tw/DOS/cp-5119-59201-113.html, accessed on 10 September 2024). Restrictions apply to the availability of these data, which were used under license for this study.

## Supplementary Materials

The following supporting information can be downloaded at: https://www.mdpi.com/article/10.3390/life14101281/s1, Table S1: International Classification of Diseases for Oncology, 3rd Edition (ICD-O-3) for cancers and International Classification of Diseases, Ninth Revision, Clinical Modification (ICD-9-CM) and Ten Revision, Clinical Modification (ICD-10-CM) Diagnosis for comorbidities.
