# Supplementary material for: Impact of Smoking on Overall and Cancer-Specific Mortality in Prostate Cancer: Elevated Risks in Older and Early-Stage Patients—A Population-Based Study
Source: Life (Basel). 2024 Oct 9;14(10):1281. doi: 10.3390/life14101281 (PMC11509152; doi:10.3390/life14101281)
Supplement: Supplementary file 1 [file life-14-01281-s001.zip › life-3228567-supplementary.pdf]

Table S1: International Classification of Diseases for Oncology, 3rd Edition (ICD-O-3) for cancers and International Classification of Diseases, Ninth Revision, Clinical Modification (ICD-9-CM) and Ten Revision, Clinical Modification (ICD-10-CM) Diagnosis for comorbidities.

	ICD-O-3	ICD-9-CM	ICD-10-CM
Prostate cancer	C61		
Comorbidity			
Diabetes mellitus		250	E08-E13
Hypertension		401-405	I10-I15
Hyperlipidemia		272	E78
